# Restricted cell functions on micropillars are alleviated by surface-nanocoating with amino groups

**DOI:** 10.1242/jcs.207001

**Published:** 2018-01-01

**Authors:** Caroline Moerke, Susanne Staehlke, Henrike Rebl, Birgit Finke, J. Barbara Nebe

**Affiliations:** 1 University Medical Center Rostock, Dept. of Cell Biology, Schillingallee 69, 18057 Rostock, Germany; 2 Leibniz-Institute for Plasma Science and Technology e.V. (INP), Felix-Hausdorff-Str. 2, 17489 Greifswald, Germany

**Keywords:** Cell–substrate interaction, Osteoblast, Ca^2+^ mobilization, mRNA expression, Microtopography, Plasma polymer nanocoating

## Abstract

The topographical and chemical surface features of biomaterials are sensed by the cells, affecting their physiology at the interface. When placed on titanium, we recently discovered osteoblasts attempted caveolae-mediated phagocytosis of the sharp-edged microstructures. This active, energy-consuming process resulted in decreased osteoblastic cell functions (e.g. secretion of extracellular matrix proteins). However, chemical modification with plasma polymerized allylamine (PPAAm) was able to amplify osteoblast adhesion and spreading, resulting in better implant osseointegration *in vivo*. In the present *in vitro* study, we analyzed whether this plasma polymer nanocoating is able to attenuate the microtopography-induced changes of osteoblast physiology. On PPAAm, we found cells showed a higher cell interaction with the geometrical micropillars by 30 min, and a less distinct reduction in the mRNA expression of collagen type I, osteocalcin and fibronectin after 24 h of cell growth. Interestingly, the cells were more active and sensitive on PPAAm-coated micropillars, and react with a substantial Ca^2+^ ion mobilization after stimulation with ATP. These results highlight that it is important for osteoblasts to establish cell surface contact for them to perform their functions.

## Abstract (precis)

**Highlighted Article:** Biomaterials, such as in medical implants, with spiky surfaces induce phagocytic processes. Plasma polymer nanocoating is able to alleviate unfavorable cell physiological reactions on microtopographies.

## INTRODUCTION

Cells are sensitive to their underlying micro- and nano-topography; in particular topographical features on microscale level offer cues that cause diverse cell responses. These responses range from altered adhesion behavior, resulting in changed integrin expression as well as signaling, to a disturbed cell function (Lüthen et al., 2005; Staehlke et al., 2015; Moerke et al., 2016). Because of steadily increasing human lifespans and the associated rising requirements placed on body parts, temporary and permanent orthopedic implants are gaining more and more relevance. An orthopedic implant should establish a mechanically solid interface with complete fusion of the material within the native bone tissue. This process is called osseointegration and can determine the successful ingrowth of the implant into the native bone, which is achieved by accelerating the onset and the rate of immediate osteoblastic cell attachment as well as proliferation (Bacakova et al., 2011). Therefore, implant surface design with its topographical as well as chemical properties, has a huge impact on osseointegration. Implant materials have to fulfill certain requirements concerning corrosion resistance and biocompatibility. Titanium (Ti) is an inert and biocompatible material and therefore commonly used as an orthopedic implant material (Bacakova et al., 2011). Osseointegration is enhanced on rougher titanium surfaces rather than on smooth ones, but it is also accompanied by changes in the cell physiology, such as the integrin expression, if the titanium surface features sharp edges (Lüthen et al., 2005; Bacakova et al., 2011). New implant design strategies pursue the development of new bioactive surfaces to evoke cellular responses that promote osseointegration (Wennerberg and Albrektsson, 2009); these include the coating of the surface to change its chemistry. In this case, coating with polymerized allylamine (PPAAm), which can be deposited by a physical low-pressure plasma process, leads to an improved osseointegration of titanium implants *in vivo* (Gabler et al., 2014), which may be caused by the enhanced cell adhesion and spreading investigated in detail *in vitro* (Rebl et al., 2012; Finke et al., 2007; Kunz et al., 2015). PPAAm is a nanometer-thin, positively charged amino-functionalized polymer layer that renders the surface more hydrophilic (Finke et al., 2007). Regular geometric micropillar topographies with the dimension of 5 µm in pillar length, width, height and spacing (P-5×5) have been used as artificial surfaces, extending the work of stochastic surface models with the advantage of constant and recurring topography variables (Lüthen et al., 2005). Previous studies have shown that osteoblastic cells mimic the underlying geometrical micropillar structure within their actin cytoskeleton, and we recently discovered an attempted caveolae-mediated phagocytosis of each micropillar beneath the cells (Moerke et al., 2016). Characteristic for this process was the dot-like caveolin-1 (Cav-1) protein and cholesterol accumulation on the micropillar plateaus after 24 h. Cav-1 and cholesterol are the major components of caveolae and are essential for the formation and stabilization of the caveolar vesicles (Parton and del Pozo, 2013). Caveolae are a specialized form of cholesterol and sphingolipid-enriched plasma membrane subdomains, called lipid rafts, distinguish themselves via the containment of the caveolin-1 protein. These specialized plasma membrane domains are involved in various cellular processes, including phagocytosis (Parton and del Pozo, 2013; Pelkmans and Helenius, 2002). The attempted caveolae-mediated micropillar phagocytosis we observed was accompanied by increased intracellular reactive oxygen species (ROS) production, reduced intracellular ATP levels and a higher mitochondrial activity (Moerke et al., 2016). A consequence of this energy-consuming process was the reduction of the osteoblast marker production, namely extracellular matrix (ECM) proteins involved in the generation of new bone tissue, for example, collagen type I (Col1) and fibronectin (FN). As a result, the cells on the micropillars showed diminished osteoblast cell function, which was also found on stochastically structured, corundum-blasted titanium with spiky elevations (Moerke et al., 2016). This indicates that the given surface microtopography also strongly affects the cell physiology in a negative sense if surface characteristics are sharp edged. In this study, we wanted to shed light on the question of whether a chemical surface modification such as PPAAm, which has a positive impact on cell spreading, adipose-derived stem cell differentiation (Liu et al., 2014) and osseointegration, can alleviate this microtopography-induced negative cellular outcome.

## RESULTS

### Nanocoating and surface characteristics

**Fig. 1.**
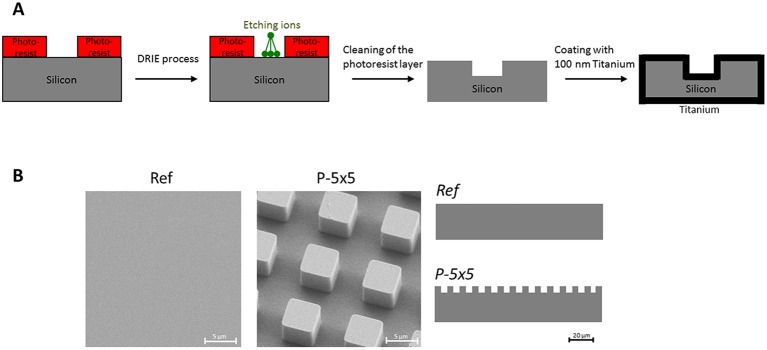
**Preparation of geometric micro-pillar model surface.** (A) Schematic illustration of the deep reactive ion etching process for the generation of micropillar topography of 5×5×5 µm (width×length×height). (B) SEM images of the planar reference (Ref) and the micropillars (P-5×5) of with a schematic side view.

**Fig. 2.**
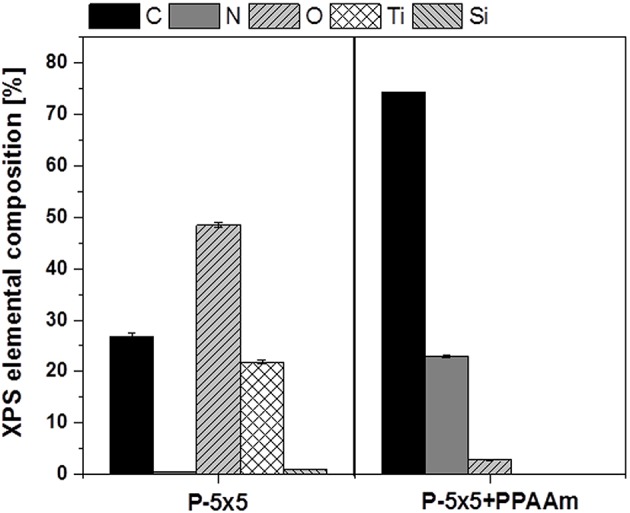
**Surface characterization of the material substrates via X-ray photoelectron spectroscopy.** Uncoated samples (P-5×5, left) and plasma polymer-coated pillars (P-5×5+PPAAm, right) were analyzed. Note that after PPAAm functionalization, titanium (Ti) and silicon (Si) are not visible, indicating a homogenous nitrogen (N)-containing layer. (XPS, Axis Ultra, Kratos).

In this study, we used substrates consisting of silicon with a final coating of 100 nm titanium. The microtopography was fabricated by deep reactive ion etching (Fig. 1). We wanted to find out whether cell functions that are restricted on the periodically microtextured samples can be alleviated by surface nanocoating with amino groups. To chemically functionalize a biomaterial surface the deposited nanolayer should have a homogenous distribution. Therefore, a surface characterization using X-ray photoelectron spectroscopy (XPS) to detect the elemental surface composition is mandatory for the detection of a pinhole-free, chemically coated layer. The density of the amino groups (ratio of NH_2_ to carbon atoms) of the plasma polymerized allylamine (PPAAm) nanolayer was ∼3% and the film thickness ∼25 nm due to the plasma deposition time of 480 s. After the PPAAm coating, no titanium (Ti) or silicon (Si) components were found on the surface (Fig. 2).

### Nanocoating and cell morphology

**Fig. 3.**
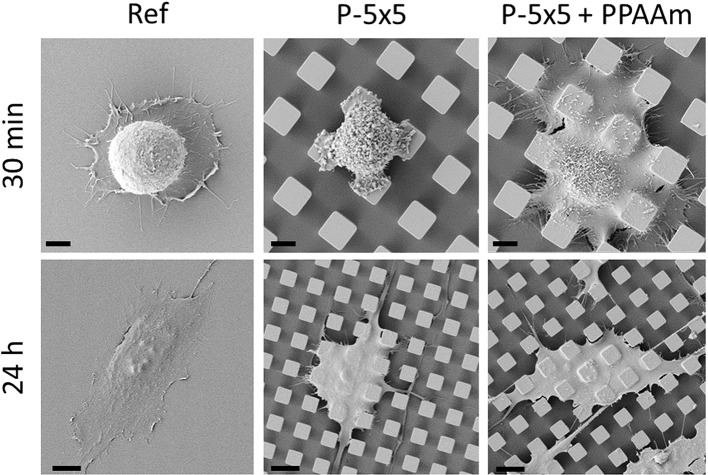
**Spreading of MG-63 osteoblasts on micropillars.** Note that after 30 min on uncoated samples (P-5×5) the cell is restricted to four pillars, whereas on plasma polymer-coated pillars (P-5×5+PPAAm) the cell area is increased dramatically. This advantage in the initial phase of occupying a surface is nearly recovered after 24 h. Top row, 3000× magnification, scale bars 4 µm; bottom row, 1000× magnification, scale bars 10 µm.

The micropillars were coated with PPAAm to enhance the cell–substrate contact by increasing the surface-occupying cell area. As shown in Fig. 3, the enhanced cell spreading after PPAAm-coating is impressive enough to be seen visually. The scanning electron microscopy (SEM) images show widely spread-out cells that are already reaching the bottom of the microtopography after 30 min on the PPAAm-coated pillar surfaces. On the uncoated micropillars, the cells exhibited a more spherical form and sit on a maximum of four pillars, whereas on PPAAm, the cells covered more than four pillars. After 24 h of cultivation, the changes were no longer as drastic as those seen after 30 min. The osteoblasts on the PPAAm-coated micropillars were still more spread out, with the micropillars more imprinted into the cells.

### The caveolin-1 and actin filaments of cells

**Fig. 4.**
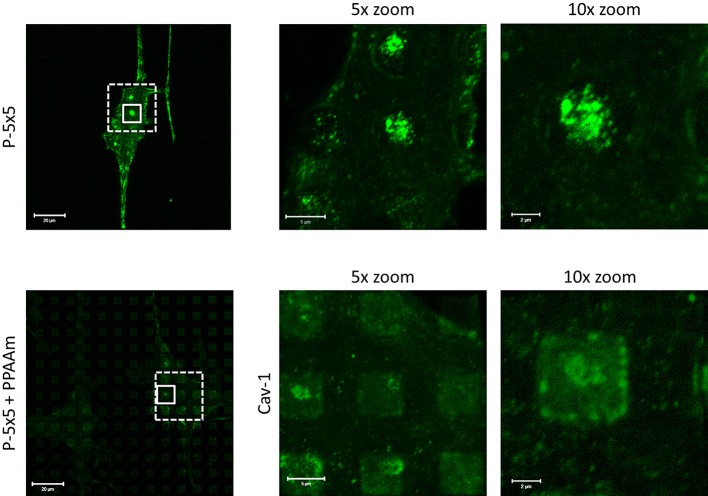
**Localization of the membrane component Cav-1 in osteoblasts on micropillars.** Note the lower levels of cluster formation for Cav-1 on plasma polymer-coated pillars (P-5×5+PPAAm) in contrast to on the control samples (P-5×5) after 24 h. Images are of *z*-stacks, magnified views are a 5× zoom of the area marked with dashed lines, or a 10× zoom of the area marked with continuous lines. Scale bars: left, 20 µm; 5× zoom, 5 µm; 10× zoom, 2 µm.

A characteristic of the attempted caveolae-mediated phagocytosis on microstructures is a clear dot-like clustering of Cav-1 accompanied with accumulated, short filaments of actin on the micropillar plateaus. The question arose, whether the osteoblasts still express the Cav-1 clusters and the clustered actin filaments on the pillar tops after coating with PPAAm. Immunofluorescence labeling reveals that there was less Cav-1 cluster formation on the P-5×5+PPAAm (Fig. 4). Cav-1 was found to be more concentrated around the pillar walls and in regions of the pillar edges after the 24 h growth period.

**Fig. 5.**
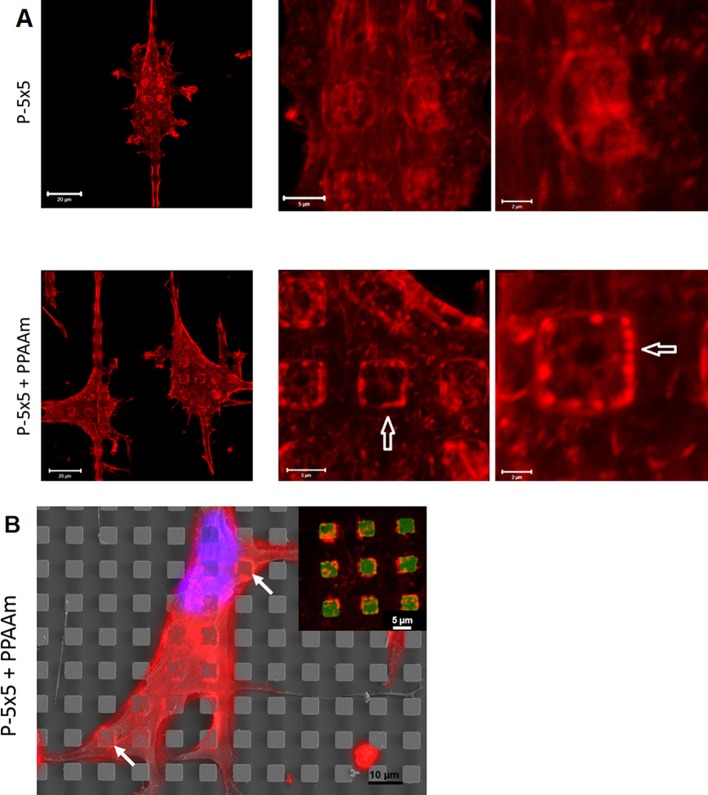
**Actin cytoskeleton of osteoblasts on micropillars.** (A) On plasma polymer-coated pillars (P-5×5+PPAAm) the actin cytoskeleton is organized in a typical ring-like structure (arrows) compared to the more clustered phenotype on controls (P-5×5) after 24 h. Images are of *z*-stacks. Scale bars: left, 20 µm; middle, 5 µm; right, 2 µm. (B) Correlative light and electron microscopy (CLEM) as a method to demonstrate the cell components – the actin filaments – in precise positions to the sample structures – the micropillars. The arrows point to the actin cytoskeleton surrounding the micropillars. Inset: the reflection mode of the microscope allows visualization of the pillars (green, false color), which are enveloped by the actin filaments (red). Scale bars: 10 µm; 5 µm (inset).

The actin cytoskeleton was accumulated in a ring-like fashion around the PPAAm-coated micropillars after 24 h (Fig. 5A). The correlative light and electron microscopy (CLEM) is a method to demonstrate the cell components (e.g. the actin filaments) as observed in our experiments, in precise positions relative to the sample topography (i.e. the micropillars). Thus, it is impressive that it can be visually seen that the pillars are enveloped by the actin filaments (Fig. 5B).

### Gene expression

**Fig. 6.**
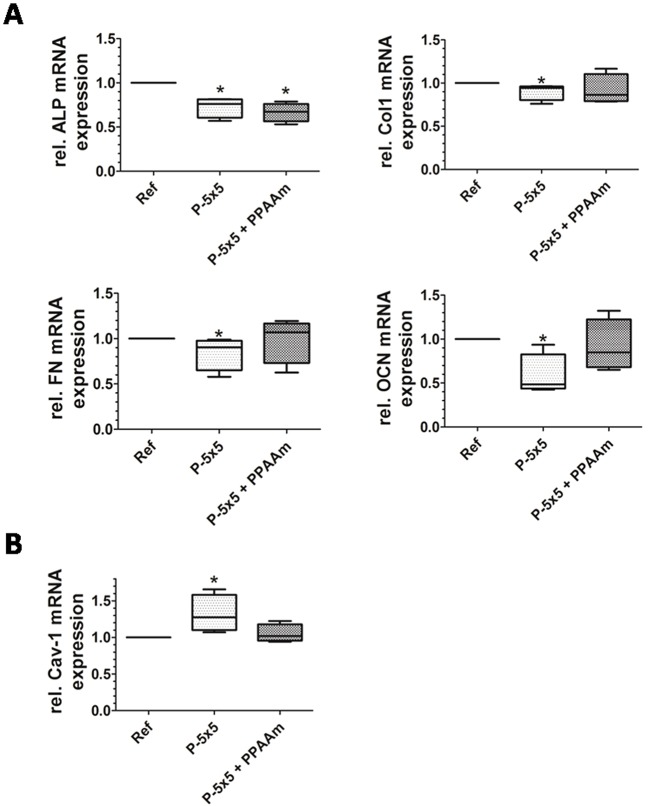
**Relative mRNA expression of osteoblasts after 24 h on micropillars.** (A) Expression levels the osteoblast marker proteins alkaline phosphatase (ALP), collagen type I (Col1), fibronectin (FN) and osteocalcin (OCN).  Note that all show restricted expression on uncoated controls (P-5×5), which is alleviated by the plasma polymer nanolayer (P-5×5+PPAAm), except for ALP. (B) Cav-1, as a membrane component that is involved in phagocytic processes is adapted to the planar reference sample if the pillars are coated with the plasma polymer. In all experiments, Ref values are normalized on 1 and *n*=4. *P<0.05 (Mann–Whitney U test).

The question was whether the PPAAm coating, with its advantage of enhanced cell–substrate contact, is able to positively influence the osteoblast marker expression. The uncoated micropillars led to significantly reduced gene expression of collagen-I (Col1), fibronectin (FN) and osteocalcin (OCN) (Fig. 6A), whereas, after PPAAm coating, these genes showed only a trend toward decrease or no difference to the planar reference. However, the gene expression of alkaline phosphatase (ALP) was significantly diminished on uncoated as well as PPAAm-coated micropillars relative to on the planar reference (Fig. 6A). In our previous experiments we observed a significantly increased Cav-1 gene expression on the micropillars (Moerke et al., 2016). The PPAAm coating influenced this process such that the Cav-1 gene expression showed only a trend toward elevation (Fig. 6B).

### Cell adhesion

**Fig. 7.**
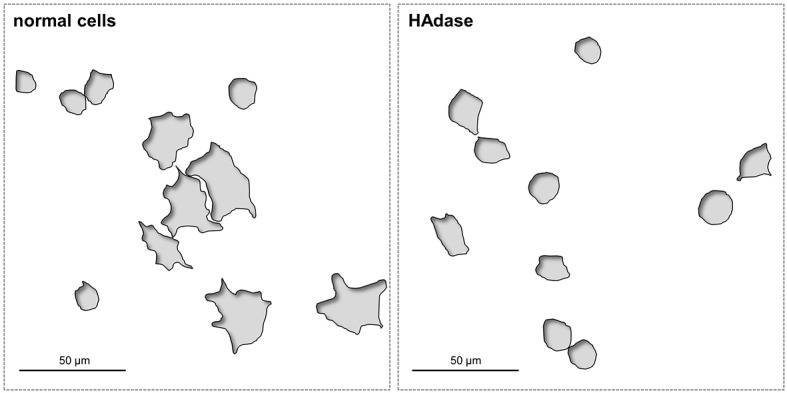
**Schema showing the importance of the hyaluronan coat of the cells on initial cell spreading.** Left: cell areas of normal osteoblasts. Right: cell areas of hyaluronidase (HAdase)-treated osteoblasts. Normal cells can spread well after 30 min on pure titanium, whereas HAdase-treated cells are hampered in their surface occupation process at 30 min. Note: the schema are presented from an original SEM image of MG-63 osteoblasts on glass-blasted titanium, HAdase 600 U treatment (for method see Finke et al., 2007).

An important question remains: how does the PPAAm nanocoating enhance the initial cell spreading? The pericellular matrix substance hyaluronan plays a key role in initial interface interactions and is essential for the first encounter of cells with the substrate (Fig. 7). We found that the initial attachment, as well as the contact-mediated spreading on titanium, is hampered if hyaluronan is cleaved by the enzyme hyaluronidase (600 U).

### Ca^2+^ signaling

**Fig. 8.**
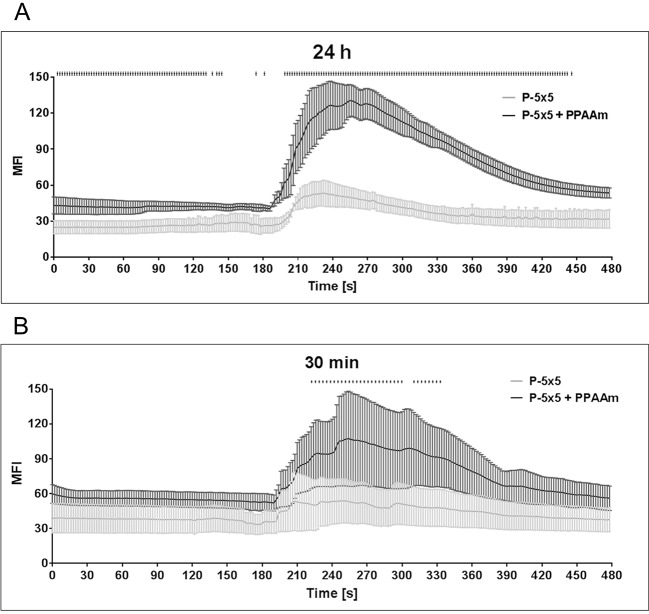
**Intracellular Ca^2+^-mobilization after ATP stimulation (180 s) in living MG-63 osteoblasts on micropillars.** (A) Influence of the plasma polymer nanolayer (P-5×5+PPAAm) on the time-dependent Ca^2+^ signal after ATP stimulation of cells allowed to adhere for 24 h. Note the substantial (significant at *P*<0.05, multiple *t*-test) increase in intracellular Ca^2+^ on P-5×5+PPAAm. In addition, the basal Ca^2+^ level is already significantly higher compared to that seen for cells on the uncoated control (P-5×5). (B) Influence of the plasma polymer nanolayer (P-5×5+PPAAm) on the time-dependent Ca^2+^ signal after ATP stimulation of cells allowed to adhere for 30 min. The increase in intracellular Ca^2+^ on P-5×5+PPAAm is clear and significant (*P*<0.05, multiple *t*-test) in the range of 244–316 s. Cells were stained with fluo-3, three independent experiments for 10 cells per time point (240 analyses). Results are mean±s.e.m.

In living osteoblasts on PPAAm, we could identify highly active cells by analysis of the Ca^2+^ mobilization capacity. The basal level of intracellular Ca^2+^ ions (0–180 s) was already significantly higher in osteoblasts established for 24 h on P-5×5+PPAAm than on uncoated P-5×5 with mean fluorescence intensity values (MFI) of 42.03±0.05 versus 26.18±0.14 (mean±s.e.m.), respectively (Fig. 8A). It is notable that these 24 h-adherent cells on PPAAm react intensively after ATP stimulation with a high increase of intracellular Ca^2+^ ions. The difference in the Ca^2+^ dynamics of fluo-3-stained cells on P-5×5+PPAAm (MFI peak at 188–246 s, 96.45±5.36; maximum MFI value, 130.35±13.11) is significant compared to cells grown on P-5×5 (MFI peak at 188–246 s, 44.43±1.84; maximum MFI value 53.36±10.78) (mean±s.e.m.) (Fig. 8A). The same cellular reactivity was found in human primary osteoblasts (HOB) after 24 h cultivation: the Ca^2+^ mobilization capacity on P-5×5+PPAAm (MFI peak at 188–246 s, 91.81±1.13; maximum MFI value, 100.46±6.47) was significantly higher compared to HOB cells on P-5×5 (MFI peak at 188–246 s: 66.62±2.52, maximum MFI value 82.49±7.89).

After 30 min of cell cultivation on P-5×5+PPAAm, MG-63 osteoblasts showed not only a more spread morphology compared to that seen on uncoated pillars as shown in Fig. 3 (30 min) but also a significantly higher Ca^2+^ mobilization after stimulation with ATP (Fig. 8B) (P-5×5+PPAAm, MFI peak at 188–246 s, 81.40±2.89, with maximum MFI value 107.30±40.90; P-5×5, 49.3±0.99, with maximum MFI value 53.88±19.1). The basal Ca^2+^ signal had already increased after 30 min of adherence on P-5×5+PPAAm (MFI mean±s.e.m.: 55.34±0.14) but this was not significant versus P-5×5 (MFI mean±s.e.m.: 37.78±0.13) as determined by a direct statistical comparison using a multiple *t*-test (Fig. 8B).

## DISCUSSION

### Cell morphology

The elemental distribution, as measured by XPS, indicated that a homogeneous, pinhole-free layer of the plasma polymer with amino functional surface groups (PPAAm) was fabricated. The deposited PPAAm coating of titanium renders the surface more hydrophilic with water contact angles for PPAAm-coated titanium versus untreated titanium (planar) of 48° and 78°, respectively (Finke et al., 2007; Liu et al., 2014), and creates positively charged surfaces (the ζ potential of PPAAm-coated titanium versus untreated titanium is +7.7 mV and −3.4 mV, respectively) (Finke et al., 2007). Owing to these surface characteristics, enhanced cell spreading after PPAAm-coating was to be seen. We observed previously that the PPAAm coating promotes the initial cell spreading after seeding of the cells and increased the cell mobility (Finke et al., 2007; Rebl et al., 2016). The PPAAm coating has proven to be a cell adhesive layer and osteoblasts can overcome topographical restrictions (e.g. contact guidance due to machined grooves) (Rebl et al., 2012, 2016). We observed that the osteoblastic cells try to internalize surface-fixed micropillars (Moerke et al., 2016) and hypothesized that cells possibly increase their cell-surface contact, because this cell surface contact is essential for the maintenance of the osteoblast-specific cell function (Saldaña and Vilaboa, 2010). Enhanced spreading as well as molding into the topography is also observed on stochastic surfaces after PPAAm coating (e.g. on machined titanium; Rebl et al., 2012; Finke et al., 2007).

### Caveolin-1 and actin filaments

The attempt by the cell to phagocytize microstructures results in a dot-like clustering of Cav-1 that is accompanied by short actin filaments on the pillar plateaus (Moerke et al., 2016). To be sure that this is a common phenomenon of cells on micropillars, we also observed the Cav-1 distribution in human primary osteoblasts and human primary fetal osteoblasts, as well as the osteoblastic cell lines Saos-2 and U-2Os and found the same accumulated pattern (Moerke et al., 2016). The Cav-1 cluster formation on the P-5×5+PPAAm after 24 h was comparable with Cav-1 on uncoated micropillars after the longer cell cultivation period of up to 96 h (Moerke et al., 2016). The accumulated actin cytoskeleton around the PPAAm-coated micropillars after 24 h was similar to that of the cells on the uncoated micropillars seen by 72 h (but not earlier) and at up to 96 h cultivation time (Moerke et al., 2016). Comparing our MG-63 osteoblastic cells with human primary osteoblasts, we previously recognized that the same accumulation of the actin filaments that was seen in MG-63 osteoblastic cells was also seen in the primary osteoblasts on pillared microstructures except that primary cells additionally have some longer actin fibers (Moerke et al., 2016).

Plasma membrane reorganization works in concert with the underlying actin cytoskeleton to mediate morphological changes and modify cell surface contacts (Head et al., 2014). Actin remodeling is especially sensitive to signals that are generated at the membrane–cytoplasm interphase (Janmey and Lindberg, 2004). The actin reorganization at the micropillar topography was shown to be characteristic for an attempt at phagocytosis. Our previous study revealed a high-energy requirement (ATP loss of 1.45-fold) that was caused by the cell attempting phagocytosis of every single pillar in a fixed position (Moerke et al., 2016). This leads to the question: why do the cells undergo the nuisance of pillar phagocytosis when this is accompanied by high energy requirements and negative cellular outcome? A possible explanation for this cell behavior would be the fact that osteoblasts are attachment-dependent cells which want to ensure the highest cell surface contact. The maintenance of the osteoblastic function relies on this contact; therefore a certain cell surface contact is needed for adequate osteoblastic cell function (Saldaña and Vilaboa, 2010). Surfaces, such as the geometrical micropillars as well as rough corundum-blasted titanium, which is a commercially used orthopedic implant design, offer the osteoblasts insufficient surface interaction area and induce internalization processes for the surface features (Moerke et al., 2016). These lead to impaired osteoblast cell function (Lüthen et al., 2005) as well as biomaterial–tissue interactions in the first phase of osseointegration. A coating such as PPAAm, which enhances the cell attachment and spreading and the interaction with the topography, has been shown to reverse these topography-induced negative effects (Moerke et al., 2017) and may also be beneficial for the improvement of commercially used rough surfaces.

### Gene expression

In our previous experiments on uncoated micropillars, the expression of osteoblast marker genes was drastically reduced after 24 h due to the attempt to phagocytize these fixed structures (Moerke et al., 2016). In our new experiments, we revealed that this restricted cell function on micropillars is alleviated by surface-nanocoating with PPAAm. However, for ALP expression the PPAAm coating was not favorable; the gene expression of ALP was significantly diminished also on PPAAm-coated micropillars. ALP is involved in the mineralization of the bone ECM. This leads to the assumption that the ALP gene expression is not induced by lessened cell surface interaction, but more by the phagocytic process itself. If a process requires high energy demands, such as phagocytosis, other energy expenditures must be abated, for example, the expression of proteins, such as ALP, which are redundant in the case that cells produce less ECM or are occupied with the establishment of sufficient cell–ECM contact (Ataullakhanov and Vitvitsky, 2002). In particular, it is to be taken into account that high energy demands are placed on osteoblasts not only during phagocytosis, but also during the production of the ECM, by expressing and secreting ECM proteins (Komarova et al., 2000). However, in experiments with polished titanium (roughness average Ra=0.19 µm) the PPAAm coating promoted the ECM expression of MG-63 cells, as is seen by the middle-to-late-stage differentiation-related ALP and bone sialo protein (BSP) mRNA production (Nebe et al., 2007).

### Cell adhesion mechanism

The pericellular matrix substance hyaluronan plays a key role in initial interface interactions (Zimmerman et al., 2002). Hyaluronan is a high molecular mass (nearly 7000 kDa) linear polysaccharide originally found in soft tissue that consists of N-acetyl-D-glucosamine and D-glucuronic acids, responsible for the negative charge of the molecule (Chopra et al., 2014). The glycosaminoglycan hyaluronan is produced by the cells itself, which causes a negatively charged spherical shell [e.g. completely enveloping chondrocytes with 4.4 µm in thickness as measured by Cohen et al. using SEM (Cohen et al., 2004; Zimmerman et al., 2002)]. Nebe and Luethen (2008) showed that a positively charged PPAAm-coating on a titanium surface could boost the initial cell adhesion, including fast formation of adaptor proteins in focal adhesions. They hypothesized that a positively charged surface provides the basis for hyaluronan-mediated attachment and spreading. Hyaluronan mediates the immediate adhesion of osteoblasts to the material surface within seconds, and the first contact is hampered on different surfaces, for example, titanium or even collagen-I, if hyaluronan is cleaved by the enzyme hyaluronidase. For instance, the cell adhesion (at 5 min) is significantly reduced by ∼5-fold if cells have to adhere to pure, polished titanium without their hyaluronan coat, and still reduced by 1.7-fold on a collagen-I-coated surface (Nebe and Luethen, 2008; Finke et al., 2007).

In our study, the energy consumption of cells could, unfortunately, not be measured due to the characteristics of the PPAAm nanolayer. PPAAm exhibits a slight autofluorescence, especially in the green channel. This would interfere with quantitative immunofluorescence analysis of these structures. Additionally, the PPAAm coating does not allow for the trypsinization of the cells in a vital state from the micropillared surfaces due to its high cell-attractive characteristics as measured previously by a spinning disc device (Fritsche et al., 2010). MG-63 cells on PPAAm were 1.5 times more resistant to shear stress compared to cells on uncoated titanium alloys (Ti6Al4V) (Gabler et al., 2014). For this reason, further experiments, such as determination of the cellular cholesterol amount, intracellular ATP as well as reactive oxygen species (ROS) generation could not be investigated in relation to uncoated micropillars. Instead, we observed the Ca^2+^ dynamics in living cells.

### Ca^2+^ signaling

Staehlke et al. (2015) demonstrated previously the negative influence of the cubic pillar topographies on Ca^2+^ signaling and dynamics compared to that seen with a planar titanium surface. Here, we could show that cells on these pillars coated with an additional plasma-nanolayer (PPAAm) with positively charged amino groups can overcome the negative influence of the pillared topography and increase their activity despite the sharp edged surface structure. This may also contribute to the positive outcome in cell function parameters typical for osteoblasts, for example, collagen-I and osteocalcin expression (see Fig. 6). The local physical microenvironment can not only modulate cell shape and mechanics (Bellas and Chen, 2014), but also the activity of cells, as shown here for intracellular Ca^2+^ levels. Although cells on pillars ‘feel’ the same microtopography (range 5 µm) the additional nanolayer (PPAAm) is decisive to trigger the cells into a more active state.

We could shed light on how plasma polymer nanocoatings can strongly alleviate the restrictions of a microtopography concerning cell functions and cell signaling. This could have importance for implant surface design and production in cases where mechanical aspects of an implant have to be considered.

### Conclusion

The present study confirms the hypothesis that osteoblasts want to create the highest possible cell surface contact to maintain their osteoblast-specific function, as displayed by the production and secretion of ECM proteins. Moreover, this analysis demonstrates that chemical surface modification with a plasma polymer nanolayer is able to attenuate the microtopography-mediated cell alterations. This PPAAm-coating process could contribute to improved cell acceptance of rough surfaces with extraordinary topographies, for example corundum-blasted titanium.

## MATERIALS AND METHODS

### Titanium surfaces

Periodically microtextured samples (size 1 cm²) with a regular cubic pillar geometry on the surface having a dimension of 5×5×5 µm in width×length×height and 5 µm in spacing (P-5×5) were used (Fig. 1). As controls, plane wafers (Ref) were employed. The samples were fabricated by deep reactive-ion etching (DRIE) (Center for Microtechnologies ZFM, University of Technology Chemnitz, Germany) on silicon (Si) wafers and coated with an additional 100 nm titanium (Ti) layer, as reported previously (Staehlke et al., 2015; Moerke et al., 2016).

### Plasma polymer nanocoating and surface characterization

Plasma polymerized allylamine (PPAAm) functionalization was performed in a low-pressure plasma process reactor V55G (plasma finish, Germany, V=60l) in a two-step procedure with the precursor allylamine (Rebl et al., 2012). The plasma deposition time for the pulsed plasma process was 480 s gross. The elemental chemical surface composition was determined by high-resolution scanning X-ray photoelectron spectroscopy (XPS). The Axis Ultra (Kratos, UK) ran with the monochromatic Al K_α_ line at 1486 eV (150 W). The spot size for high-resolution measurements was limited to 250 µm. Spectra were recorded at a pass energy of 80 eV (Finke et al., 2007).

### Osteoblast cell culture

The human osteoblast-like cell line MG-63 (American Type Culture Collection ATCC®, CRL1427™) was used throughout the experiments. MG-63 cells have an integrin subunit profile similar to that of primary human osteoblasts and were considered applicable for studying initial cell attachment processes to surfaces (Czekanska et al., 2012). In our own experiments, we have found similarities in the formation of β1- and β3-integrin receptors in MG-63 osteoblasts grown on structured titanium (e.g. glass-blasted, corundum-blasted) that were comparable with human-derived primary osteoblasts (Lüthen et al., 2005). The cells were grown in Dulbecco's modified Eagle's medium (DMEM, Life Technologies GmbH, Darmstadt, Germany) with 10% fetal calf serum (FCS) (Biochrom FCS Superior, Merck KGaA, Darmstadt, Germany) and 1% gentamicin (Ratiopharm GmbH, Ulm, Germany) at 37°C in a humidified atmosphere with 5% CO_2_. Human primary osteoblasts (HOB, C-12720, PromoCell GmbH, Heidelberg, Germany) were cultured in osteoblasts growth medium with SupplementMix (PromoCell) and 1% antibiotic-antimycotic (Anti-Anti 100×, Life Technologies). In the experiments, 15,000 cells/cm² were seeded onto the samples placed in NUNC 4-well dishes (Thermo Fisher Scientific, NUNC GmbH & Co. KG, Langenselbold, Germany). Before use, the uncoated titanium arrays were washed in 70% ethanol for 10 min and rinsed three times in phosphate-buffered saline (PBS) (Sigma Aldrich, Munich, Germany).

### Real-time quantitative PCR for mRNA expression

Total RNA was isolated after 24 h cell growth on the samples using a NucleoSpin® RNA II kit (Macherey-Nagel GmbH & Co KG, Dueren, Germany), which includes a step for the elimination of any genomic DNA by DNase (Macherey-Nagel) treatment. The purity and quantity of the resulting RNAs were determined via measurement of the absorbance at 280 nm and 260 nm with the Nano Drop 1000 (Thermo Scientific). 50 ng of total RNA was used for first-strand cDNA synthesis by using Superscript® II reverse transcriptase and random primers (Invitrogen AG, Carlsbad, CA, USA). The real-time quantitative polymerase chain reaction (RT-qPCR) was performed with TaqMan® Universal PCR Master Mix and TaqMan® gene expression assays for alkaline phosphatase (ALP) (Hs00758162_m1), caveolin-1 (Cav-1) (Hs00971716_m1), collagen type 1 (Col1) (Hs0016404_m1), fibronectin (FN) (Hs00900054_m1), glyceraldehyde-3-phosphate dehydrogenase (GAPDH) (Hs99999905_m1) and osteocalcin (OCN) (Hs01587813_g1) (all Applied Biosystems by Life Technologies GmbH, Darmstadt, Germany) following the manufacturer's instructions. The TaqMan® PCR assay for each target gene was performed in triplicate for four independent experiments. The PCR was performed with a RT-PCR Applied Biosystem 7500 machine and the data were collected and analyzed by the 7500 System SDS Software (Applied Biosystems). Each expression was calculated relative to GAPDH (ΔCt) and then relative to the references (ΔΔCt).

### Immunofluorescence staining

MG-63 osteoblastic cells were cultured on the titanium arrays for 24 h, washed three times with PBS and then fixed with 4% paraformaldehyde (PFA) (10 min; room temperature) (Sigma-Aldrich). After washing three times with PBS, the cells were permeabilized with 0.1% Triton X-100 (10 min, room temperature, Merck), washed again three times with PBS and blocked with 2% bovine serum albumin (BSA) (Sigma-Aldrich) in PBS (30 min, room temperature).

For actin filament staining, cells were incubated with phalloidin coupled to tetramethylrhodamine (TRITC) (5 µg/ml in PBS, Sigma-Aldrich). Nuclei were stained for 10 min with Hoechst 33342 dye (1 mg/ml; Sigma Aldrich, 1:2000 in PBS).

Caveolin was stained with the polyclonal rabbit anti-caveolin-1 (cat. no. 3238, New England Biolabs GmbH, 1:400) as primary antibody for 1 h at room temperature followed by the secondary antibody anti-rabbit-IgG conjugated to Alexa Fluor 488 (Life Technologies, diluted 1:200 in PBS) applied at room temperature for 30 min. The samples were embedded with Fluoroshield^TM^ mounting medium (Sigma-Aldrich).

### Confocal laser scanning microscopy

Fluorescent cells were observed using an inverted confocal laser scanning microscope (LSM 780; Carl Zeiss AG, Oberkochen, Germany) with a ZEISS oil immersion objective (C-Apochromat 63). For image acquisition, the ZEISS Efficient Navigation (ZEN) 2011 SP4 (black edition) software was used. All images were displayed as three dimensional *z*-stacks of 13 slices with an interval of 1 µm. High-resolution images of the actin cytoskeleton in Fig. 5B (inset) were recorded with the LSM 410 (Carl Zeiss AG), equipped with a 63× oil immersion objective (1.25 oil/0.17). For visualization of the corresponding substrate region, the reflection mode was used and the pillars were false colored in green.

### Correlative light and electron microscopy

For correlative microscopy, the fluorescence images of the actin stained, non-embedded samples were acquired with the upright microscope AxioScopeA1 (Carl Zeiss) equipped with a 63× water objective. The calibration of the samples and image overlay was performed with the software AxioVision v. 4.8.2 (tool: shuttle and find). Calibration of the samples was accomplished by using the edges of the sample as point of reference. Afterwards, the samples were prepared for scanning electron microscopy as described below.

### Scanning electron microscopy

Cells were grown for 30 min, or 24 h on the samples, washed with PBS three times and then fixed with 2.5% glutardialdehyde (cat. no. 814393, Merck KGaA, Darmstadt, Germany) for 1 h at room temperature, dehydrated through a graded series of ethanol (30, 50, 75, 90 and 100% for 5, 5, 15, 10 min and twice for 10 min, respectively) dried in a critical point dryer (K 850, EMITECH, Taunusstein, Germany) and then samples were sputtered with gold for 100 s (layer of ∼20 nm) (SCD 004, BAL-TEC, Wetzlar, Germany). Scanning electron microscopy (SEM) observations were performed with a field emission (FE)-SEM (ZEISS Merlin VP compact, Carl Zeiss AG). The recovering of the desired areas for correlative microscopy was performed with the software AxioVision v. 4.8.2 (tool: shuttle and find) after sample calibration.

### Ca^2+^ ion imaging in living osteoblasts

Fluo-3 staining of suspended cells was performed on trypsinized MG-63 cells that were washed in PBS (containing Ca^2+^ and Mg^2^^+^) and afterwards stained with the Ca^2+^ ion indicator fluo-3/acetoxymethylester (fluo-3/AM, 5 µM, Life Technologies Corporation, Eugene, OR) in hypotonic HEPES buffer (Staehlke et al., 2015). Subsequently, the osteoblasts were collected in isotonic HEPES buffer and seeded onto the micropillars (with or without PPAAm). After 30 min cultivation, the samples were turned upside down in a ibidi µ-dish (ibiTreat, Ø 35 mm; ibidi GmbH, Martinsried, Germany) and analyzed in the confocal microscope.

Fluo-3 staining of adherent cells was performed after a 24 h cultivation of MG-63 cells on micropillars (with or without PPAAm). The cells were marked with fluo-3/AM as described above. For the analysis, the osteoblasts were cultured in isotonic HEPES buffer (Staehlke et al., 2015) as described for suspended cells.

Living cell observations were performed as follows. The analysis of intracellular Ca^2+^ mobilization was performed on the confocal microscope LSM 780 (Carl Zeiss) equipped with an argon-ion laser (excitation at 488 nm) using a Zeiss 40× water immersion objective (C Apochromat, 1.2 W Korr M27). The measurement of the mean fluorescence intensity (MFI) of the global Ca^2+^ signal was carried out by Zen2011 (SP4, black) software (Carl Zeiss) with the mode ‘time series’. Images (512×512 pixels) from at least three or four (30 min and 24 h, respectively) independent experiments for 10 cells per time point (for 240 cycles) were measured at full pinhole (13.5 µm section) and the same settings. During recording of the time series over 240 cycles (2 s each) adenosine 50-triphosphate (ATP, 10 µl, SERVA Electrophoresis GmbH, Heidelberg, Germany) was added after the 90th cycle for stimulating the intracellular Ca^2+^ mobilization. The evaluation of fluorescence signals in defined areas of individual single cells over time was carried out by using ZEN2 (blue edition, Carl Zeiss) using the function ‘mean ROI’.

### Statistical analysis

Statistical analyses were carried out with GraphPad Prism5 software (GraphPad Software Inc., La Jolla, CA). Results are presented in box plots with medians, quartiles and an interquartile range (IQR) with whiskers representing 1.5×IQR. Data analyses were performed with a Mann–Whitney U test. For Ca^2+^ analyses, the multiple *t*-test was used as well, and the time-dependent data are presented as mean±s.e.m. *P*<0.05 was considered to indicate significant differences.
